# DADS: Depression Among Dads: A Multi-Centric Cross-Sectional Study on the Prevalence, Awareness, and Risk Factors of Paternal Postpartum Depression in Pakistan

**DOI:** 10.1192/bjo.2026.11189

**Published:** 2026-06-30

**Authors:** Amna Zaheer, Faheem Vellekkat, Vijay Chavada, Hira Ahmad, Sadiq Naveed

**Affiliations:** 1 Liaquat National Hospital and Medical College, Karachi, Pakistan; 2 Indira Gandhi Medical College and Research Institute, Puducherry, India; 3 NHS Lothian, Edinburgh, United Kingdom; 4 University of Connecticut, Connecticut, USA

## Abstract

**Aims:**

Paternal postpartum depression (PPD) is an emerging concern globally but remains under recognized in low-resource and patriarchal societies. In Pakistan, sociocultural stigma and lack of mental health infrastructure may exacerbate this burden. This study aimed to assess the prevalence of paternal PPD, identify associated risk factors, and evaluate awareness among Pakistani fathers.

**Methods:**

A cross-sectional, multicentre study was conducted across three provinces of Pakistan, enrolling 475 fathers of infants under one year of age. Participants completed the Beck Depression Inventory (BDI) and a structured questionnaire covering demographics, reproductive history, awareness of PPD, and psychosocial stressors. Statistical analyses included t-tests, chi-square tests, ANOVA, and Pearson correlation.

**Results:**

A BDI score ≥10, indicating mild or greater depressive symptoms, was observed in 70.1% of participants, while 10.1% had severe symptoms suggestive of clinical depression. Only 19.4% of fathers were aware that men could experience PPD. Significant associations were found between paternal depression and maternal PPD (p=0.002), adverse life events (p=0.003), and lack of family support (p=0.045). Age showed a weak positive correlation with depression severity (r=0.19, p<0.001). No significant associations were observed for occupation, number of children, awareness, or access to leave policies. A majority (94.7%) reported no access to paternal mental health services.

**Conclusion:**

This is the largest study to date on paternal PPD in Pakistan, highlighting a substantial burden of depressive symptoms among new fathers. Cultural stigma, poor awareness, and lack of support structures contribute to under-recognition and under-treatment. Multi-level interventions to address paternal PPD, including but not limited to screening strategies, paternal-inclusive care, and public education are urgently needed

